# A Review of Integration Strategies to Support Gene Regulatory Network Construction

**DOI:** 10.1100/2012/435257

**Published:** 2012-12-27

**Authors:** Hailin Chen, Vincent VanBuren

**Affiliations:** Department of Medical Physiology, Texas A&M HSC College of Medicine, Temple, TX 76504, USA

## Abstract

Gene regulatory network (GRN) construction is a central task of systems biology. Integration of different data sources to infer and construct GRNs is an important consideration for the success of this effort. In this paper, we will discuss distinctive strategies of data integration for GRN construction. Basically, the process of integration of different data sources is divided into two phases: the first phase is collection of the required data and the second phase is data processing with advanced algorithms to infer the GRNs. In this paper these two phases are called “structural integration” and “analytic integration,” respectively. Compared with the nonintegration strategies, the integration strategies perform quite well and have better agreement with the experimental evidence.

## 1. Introduction

### 1.1. Conventional Strategies of Building GRNs

Biological functions comprise numerous reactions at all levels of biological organization, including cells, tissues, organs, and body, and interchange with the environment. Overall, every life phenomenon found in this multilevel system is supported through many reactions interconnecting with each other to compose the orchestra of life. It is, therefore, crucial to have a systematic perspective in biomedical research. To gain an overview of such a complex system, we can visualize it in the form of a network. For instance, protein-protein interactions, metabolic reactions, and genetic regulations correspond respectively to a protein-protein interaction network (PPI), metabolic network, and gene regulatory network (GRN), which are subnetworks of the complex multi-level system. In the representation of a network, nodes typically correspond to molecules, while edges represent the relationships between nodes. In the study of biological networks, GRNs are one of the most popular models, especially in the field of development. Developmental GRNs provide important clues to elucidate the temporal and spatial dynamics of gene expression during development. The use of sea urchin and *Drosophila* has led to some of the greatest successes in studying developmental GRNs to explain complex developmental processes [1, 2]. Traditionally, the first step is to identify putative regulatory genes through genome-wide screening, such as expression microarrays, across distinct temporal and spatial states. Quantitative PCR is used afterwards to verify specific expression patterns [3]. Amazingly, the gene repertoire used in the control of development is relatively conserved across species, and thus regulatory genes can be identified by sequencing-based homology alignments [4]. As a central objective of modeling developmental GRNs is to identify the epistatic relations among these regulatory genes, the second step is to define experiments to perturb/activate the system and examine the responses via loss-of-function and gain-of-function experiments [3]. In a sea urchin GRN study, perturbation with morpholino-substituted antisense oligonucleotides (MASOs) was the main approach [5]. Rescue experiments are also an important part of this step. Finally, by assembling findings from many individual experiments, investigators may establish the developmental GRN. Validation of the established GRN can be accomplished precisely via mutagenesis of regulator binding sites for their target genes to observe the abolishment of the regulatory effect [6, 7].

Elucidation of gene regulation in the endomesoderm specification in the sea urchin and in the development of *Drosophila* embryos provides potent examples of the type of complexities revealed by the study of GRNs. In the sea urchin embryo, *blimp1* is autorepressive when its product accumulates to high levels. At the same time, it provides a required input for *Wnt8* expression, which produces a positive feedback effect for *blimp1* via inducing *Tcf* to activate *blimp1* expression. *Wnt8* can infect the adjacent cells/territories with this circular bioinformation flow via diffusion. This flow is terminated due to *blimp1* autorepression [8]. In the early development of the *Drosophila* embryo, *Snail* repressor activates the synthesis of *Delta* ligand in the ventral mesoderm via repressing the transcription of *Tom*, an inhibitor of the *Delta*, which is called a double-negative gate. *Delta* triggers *Notch* signaling in the adjacent cells via diffusion. However, transcription of the *Notch* signaling target genes is repressed by the intraterritorial *Snail* repression in the ventral mesoderm itself. An exactly parallel mechanism causing transcriptional alternation inter-territorially is also found in the sea urchin skeletogenic mesoderm [1, 2]. Despite such accomplishments, there is still a large portion of the overall GRN in animal models that has not been defined. The laborious approach to elucidating GRNs from experiments for every node and every edge produces reliable biological information as prior knowledge to support novel findings. However, due to the complications in GRNs as discussed above, elucidating the complete GRN of complex eukaryotic organisms with respect to the whole genome would be extremely difficult using this strategy, as much time and labor are required even for just one conditional state. The strategy described above is the bottom-up approach of network construction. Computational strategies offer a top-down approach to network construction that complements what is described above.

### 1.2. Computational Strategies for Building GRN

#### 1.2.1. Nonintegration Strategies

During this blooming period of biomedical research, high-content experimental data is fuelling systems biology research, such as GRN construction at the genome-wide scope. For example, expression microarrays that can detect the relative abundance of gene transcripts by comparing two or more biological samples are commonly used for GRN construction. The new approaches provide a perspective on the global molecular interactions that bridge the gap between the external signal and internal response. There are several popular algorithms being used to construct GRNs from expression data (reviewed in [9]).

In the graphical representation of GRNs, nodes typically represent genes corresponding to the transcription factor proteins or target genes, while edges represent the regulations between the transcription factors and their targets. Boolean networks describe each element as a variable with the value 0 or 1 to represent the state of the element as “off” or “on,” respectively. A Boolean network *G*(*V*, *F*) is defined by a set of nodes corresponding to genes *V* = {*x*_1_,…, *x*_*n*_} and a list of Boolean functions *F* = (*f*_1_,…, *f*_*n*_) describes how genes in the network change their state (on or off) from one time point to the next. The future state of an element is completely determined by the states of other elements (regulators) by means of the underlying logical Boolean functions. 

Second, Bayesian networks model the biomedical network with a *directed acyclic graph*. “*Directed*” means that there are arrows to indicate causal influences, and “*acyclic*” means that causal loops are prohibited. For each element, a conditional distribution *P*(Xv ∣ parents(Xv)) is defined through the application of the conditional probability table (CPT), where parents(Xv) denotes the variables corresponding to the regulators of this element. Thereafter, an optimization approach is applied, with the Bayesian information Criteria (BIC) optimized to infer the best fitting network model among a finite set of models. 

In a third alternative, differential equations extract the network from high-throughput experimental data through taking the instantaneous concentration of each element into consideration. The instantaneous concentration of each element is completely determined by the concentration (*x*_*n*_) of other elements involving a regulation function. 

*Differential equation modeling*:

(1) $$\begin{array}{c} \frac{dx_{i}}{dt}=f_{i}\left(x_{1},\ldots ,x_{n},t\right). \end{array}$$

In a fourth alternative, coexpression is used to model GRNs based on co-variance analysis. However, the comparison between the covariances from datasets having different scales would be difficult. The Pearson correlation coefficient addresses this difficulty. It measures the coexpression between any two elements across a series of states resulting in the value with the range from −1 to 1, which allows networks to be established based on a certain threshold for the magnitude of the correlation. 

Finally, Mutual Information (MI) offers another approach to modeling GRNs based on the probability theory. The mutual dependence of any two elements in the network is measured using MI. It is reported that MI outperforms the correlation in some studies [10, 11]. Using a reasonable threshold, networks will be accurately constructed. Context likelihood of relatedness (CLR) [10, 12], MRNet (maximum relevance/minimum redundancy network) (R package), and ARACNE (algorithm for the reconstruction of accurate cellular networks) [11, 13] are the three representative strategies of network construction applying MI. Numerous approaches to GRN construction have been developed using various combinations of the five main approaches described above.

#### 1.2.2. Motivations for an Integration Strategy

The most popular algorithms contributing to the construction of GRNs from genomic expression data were described above. However, each of them has certain drawbacks. The Boolean algorithm assigns each variable a binary value, which could omit important information of continuous variables. Bayesian network construction is promising for representing and inferring causal relationships, but this strategy is only effective for the construction of small GRNs, due to the superexponential increase in the algorithm running time for large networks. The differential equation algorithm requires knowledge of the equation of dynamics and parameter estimation to optimize the GRN model against real data. However, deriving an appropriate equation of dynamics remains a challenge. Furthermore, solving a differential equation system of any realistic complexity is difficult. As to the correlation and mutual information algorithms, manually setting appropriate thresholds without a principled reference poses difficulties. Strategies applying algorithms with these drawbacks are not satisfying; therefore, it motivates us to improve the computational strategies. New strategies continue to be developed against those difficulties. It is a great challenge to refurbish algorithms to improve GRN construction using genomic expression data. Improvements are difficult to obtain algorithmically; however, the integration of multiple types of genome-wide datasets with literature-based information of regulation as prior knowledge is a straightforward alternative to offer improvement. Generally, in the computational GRN construction methods mentioned above, only genomic expression data like microarray data is used to produce the desired network applying one of the algorithms described [10, 11]. Based on a straightforward intuition that more relevant information generates better confidence for making correct predictions, we are optimistic about the prospects of making improvement by data integration. We have increasing availability of genome-wide data with respect to every aspect of biology, genomic expression data, genome sequences, proteomic data, genome-wide protein-DNA binding site data [14], genomic SNPs, and high-content data collections created from various types of biological or pathological research objectives. Therefore, with reference to the literature-based information of regulation as the prior knowledge and the multiple types of genome-wide datasets available as analyzable data, an integration strategy can offer an excellent opportunity for elucidating complete GRNs. 

## 2. Integration Strategies for Building GRNs

### 2.1. Sources for Integration

The past few decades were an age of rapid progress in the development of biomedical science. Numerous advanced technologies along with well-founded theories lead the way for new findings in industrial and academic biomedical research. For example, biomedical investigators have developed genomic expression by microarray, rapid genome and microbiome sequencing, proteome definition by mass spectrometry, genome-wide protein-DNA binding site definition by ChIP-seq, genomic SNP identification by SNP array, and high-content knowledge by literature mining. Overwhelmed with such impressive quantity of genome-wide achievements, we are encouraged to apply strategies to make good use of them intuitively, such as integrating them properly for GRN construction. First we need to take stock of the status of the biomedical sources that are available to us. 

**Figure 1:**
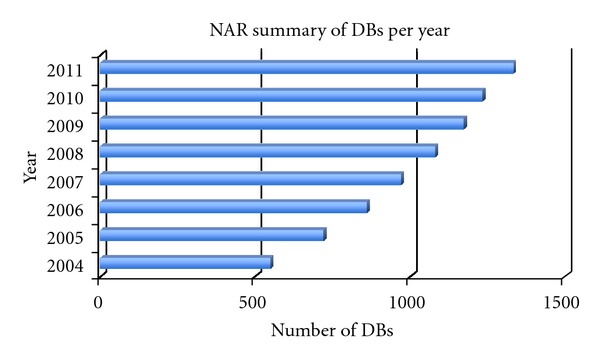
This is the database (DB) summary from NAR database issues. Each bar represents the total number of databases identified by NAR that year.

It is difficult to summarize all the biomedical sources as most sources are scattered in distinct research papers. We will focus our attention on databases, as they are an effective form of rearranging and storing sources for specific objectives. Nucleic Acids Research (NAR) summarizes the biomedical database status each year (Figure 1) (http://nar.oxfordjournals.org/). Here is a table (Table 1) summarizing some genome-wide databases popular in the research of systems biology.

### 2.2. Structural Integration

A large number of genome-wide sources are available that have not been fully leveraged to infer novel GRNs. Before entering into a discussion of the analytic algorithms for integrating multiple genome-wide datasets for GRNs construction, we must first address the challenge of extracting the desired datasets from the ocean of biomedical sources. Structural integration retrieves desired datasets from multiple heterogeneous sources to facilitate querying the data for further analytic integration. There are many sophisticated approaches being used for structurally integrating target datasets through programmatic extraction and recombination. Overall, these approaches to structural integration can be divided into three general categories: warehouse integration, mediator-based integration, and navigational integration [15].

Before discussing the approaches to structural integration in the following Sections 2.2.1–2.2.3, we will finish this section with a discussion of some key defining characteristics of structural integration.

**Variety of Data**

This describes the typical data that can be integrated and includes high-throughput datasets, molecular structures, molecular interactions, molecular pathways, Gene Ontology annotation, and disease characteristics, hence *vertical integration* is the aggregation of *semantically similar* data from multiple heterogeneous sources, while *horizontal integration* is the composition of *semantically complementary* data from multiple heterogeneous sources [15].

**Heterogeneity of Descriptive Terms**

Semantics is the study of the relation between form and meaning. Each source of data or knowledge may refer to the same semantic concept or field with its own descriptive term or identifier, which can lead to a semantic confusion between the many sources. Conversely, some sources may use the same term to refer to the different semantic concepts. Semantic mapping is indispensable in order to match descriptive terms or identifiers among multiple heterogeneous sources or between the sources and the objective integrated datasets.

**Heterogeneity of Naming and Identity**

One major hurdle in current data integration efforts is the issue of naming and identity such that a variety of aliases (e.g., synonyms for gene symbol) exist for many genes, proteins, and keywords. Alias mapping through lookups is critical for retrieving desired data from multiple heterogeneous sources.

#### 2.2.1. Warehouse Integration

Warehouse integration arranges desired datasets from multiple sources into a local warehouse (e.g., a local database) before querying, through loading the required data from distinct sources and converting them into standard formats before being stored locally. Relying less on the Internet connectivity to access data limits the impact of various problems such as access restrictions, network bottlenecks, low response times, and the occasional unavailability of sources. Moreover, using local warehouses allows for improved accuracy, efficiency, and flexibility for the subsequent query as it is performed locally. However, this integration has an important drawback of the overall system maintenance. It is expensive to have the warehouse updated regularly to reflect those modifications of heterogeneous external sources [15, 16]. Furthermore, since the data retrieved and stored in the warehouse will eventually be converted into the warehouse-specific format every time the warehouse is updated, the semantic structure of the warehouse database may need to be reformatted often. 

NCBI, the UCSC Genome Browser [17], and EMBL-EBI (http://www.ebi.ac.uk/) are three representative data warehouses. Given the appeal of these resources, efforts are increasingly made to improve the warehouse strategy against its drawbacks. The GeNS platform is one of the efforts to improve the efficiency of database maintenance. GeNS is a biological data integration platform for warehouse integration [18]. Representative databases were selected to cover a broad area of biomedical research when constructing the GeNS database. This warehouse accommodated the data from EMBL-EBI, UniPort (Swissport and TrEMBL), ExPASy (PROSITE and ENZYME), NCBI (Entrez, Taxonomy, Pubmed, RefSeq, GeneBank, and OMIM), Biomart, ArrayExpress, InterPro, Gene Ontology, KEGG (genes, pathways, orthology and drugs), and PharmGKB (genes, drugs, and diseases). A loader application responsible for converting the corresponding data from each source database into the format compatible with GeNS schema was designed to coordinate tasks such as alias mapping. In order to overcome the difficulty of maintenance, a general schema and a specific schema were both developed in GeNS. To physically store the data, a general model (general schema) that certified the framework of the database was used, while supporting this general model with a concrete meta model (specific schema) where all the entities and relations from a specific contributing database were specified locally [18]. Therefore, the addition/modification of databases into this warehouse needs modification in the meta model only, rather than in the general model. 

#### 2.2.2. Mediator-Based Integration

Mediator-based integration retrieves desired datasets from multiple heterogeneous sources at the time of querying through query translation, as opposed to the data translation that is manifested at the time of database creation in warehouse integration [15, 16]. The mediator, or core of the query translation, is an interface responsible for reformulating a query given by the user into the queries accommodating the local schemas of the underlying data sources via a single mediated schema defined by the mediator-based integration platform. Therefore, a mapping is required in the mediated schema to capture the semantic relation or the identity alias' relation between the sources and the given query, which thus allows the query made by a user to be translated via the mediator into the appropriate queries onto the individual sources. This correspondence mapping is a crucial step in creating the mediator, as it will influence the query reformulation and the addition of new sources to or the removal of the old sources from the integrated system. 

There are two main approaches for establishing the mediator, global-as-view (GAV) and local-as-view (LAV) [15, 16]. The GAV has the mediator that translates the given queries directly into the formats of the source queries. The LAV has the format of query in every source defined into the common format of mediation, which is defined by the mediator via a wrapper. Therefore, each local source needs a wrapper component that exports a view of the local data into a common format of mediation via mediated schema. Since the mediator-based integration retrieves data at the run-time of querying, the problems such as access restriction, network bottlenecks, low response time, and the occasional unavailability of sources may occur. However, since the queries are performed in the real-time fashion, there is no special need of system maintenance via manually updating the databases. More specifically, LAV makes it very simple to add or to remove sources, while for GAV the addition or removal of sources is much more difficult, as it requires a modification of the mediated schema on the correspondence mapping. 

The mediator approach is a very popular approach of data integration. Platforms like K2, TAMBIS, Discovery-Link, and BACIIS are all designed based on this approach. In the Discovery-Link platform (http://www.redbooks.ibm.com/abstracts/sg246290.html/), the source-specific wrapper symbolizes its data sources for further integration. 

#### 2.2.3. Navigational Integration

To extract the desired datasets, navigational integration follows the workflow in which the query outputs from a source are redirected as the query inputs to the next resource until the requested information is reached [15, 16]. It resembles the nature of the web in the context of increasing number of data sources, and it, therefore, frees users from manually browsing several web pages or data sources in order to obtain the desired datasets. However, the drawbacks of the navigational integration are similar to those of the mediator-based integration, such as access restrictions, network bottlenecks, low response times, and the occasional unavailability of sources. Additionally, the time and effort required to build the correspondence mapping are still costly. 

Examples of this approach are Entrez and DiseaseCard databases. DiseaseCard [19] is a web-based collaborative service that aims to comprehensively integrate genetic and medical information, including the information of rare genetic diseases. 

#### 2.2.4. Choosing a Method for Structural Integration

Here is a brief comparison (Table 2) that summarizes the features of different structural integration approaches of extracting desired datasets from the ocean of biomedical sources. 

The main purpose of the structural integration in most cases is to compile all available information for specific objectives to prepare for arbitrary analytic integration according to the user interest. 

An ideal integration schema should have the following characteristics.

1. Efficient. It can optimize the time that users need to finish the query. One of the recent ideas is to build semantic webs.
2. Easy to maintain.
3. Stable.
4. System performance metrics. It is critical for an integration system to study source statistics in order to refine the query plans and improve the overall functionality and performance of the system. The essential statistics that should be learned are the coverage of sources, the average response time, the cost, and the overlap between sources [15].
5. High quality. The data integrated are extracted from various heterogeneous sources, having different degrees of quality. For example, compared with the old data, new data from improved technologies may have better quality; also, compared with computationally predicted data, the experimental data is expected to have better quality. Quality varies within heterogeneous data sources, and some effort to account for these differences should be considered in the data integration strategies.
6. Automated. the disciplines of operational optimization and machine learning should be applied for an effective automation program.

### 2.3. Analytic Integration

Along with the desired datasets extracted from multiple heterogeneous sources through structural integration, analytic integration is performed to infer GRNs via data integration algorithms applied to the desired datasets. The integration algorithm is, therefore, an essential ingredient for optimizing GRN construction. In contrast with the algorithms described in the above section, the integration algorithm needs to be capable of dealing with multiple types of data simultaneously. As a result, heterogeneous data should merge smoothly regardless of the differences in data types. As we discussed previously in Section 1, many types of genome-wide datasets could contribute to GRN construction. In the following discussion of the analytic integration for GRN construction from multiple types of genome-wide datasets or with reference to prior knowledge, there are three main schemas to consider: naïve Bayesian applications, supervised learning, and network topology applications. Each of these schemas represents a distinct approach to analytic integration, yet each can be applied to multiple categories of hypothesis inference, such as transcriptional regulation, protein-protein interaction, and gene-disease association. 

#### 2.3.1. Naïve Bayesian Applications

The Bayesian schema applying the naïve Bayesian is specified in the biological context: if association of two molecules occurs across multiple heterogeneous sources, there is an increased likelihood that they have a strong connection that may, for example, include a productive regulation or an indispensable physical interaction. Therefore, the functional importance of the pairwise connection is evaluated through its incidence across the multiple sources. And many types of genome-wide datasets, such as genomic expression and phylogenetic profiles, will contribute to the perceived functional importance of the pairwise connections in the genome-wide scope. Therefore, a scoring system is then applied to evaluate the functional importance of the pairwise connections in the genome-wide scope to gain insight about the confidence of the inferred GRNs or PPIs. Two successful examples with naïve Bayesian applications are described below. 

The STRING web application was designed to infer the PPI via integrating multiple types of genome-wide datasets. It was primarily constructed from the integration of three genome-wide datasets, including phylogenetic profiles, a database of transcription units, and a database of gene-fusion events [20–24]. Phylogenetic profiles are derived from the evolutionary tree. During evolution, functionally linked proteins tend to be either preserved or eliminated in new species simultaneously. This property of correlated evolution is leveraged in the STRING database by characterizing each protein via its phylogenetic profile that records the presence or absence of an orthologous protein in every known genome. Those proteins having matching profiles have a strong tendency to be functionally linked. Transcriptional units (operons) are extracted from a number of genomes through identifying the conserved gene clusters. The protein products of the genes in transcriptional units are hypothesized to be functionally linked with each other. Gene-fusion events can be interpreted by the example that the interacting proteins GyrA and GyrB subunits of *E. coli* DNA gyrase are orthologs of a single fused chain (topoisomerase II) in yeast; thus, the similarities of GyrA and GyrB to some segment of topoisomerase II might be used to predict their functional interaction in *E. coli*. STRING was developed as a multi-dimensional integration interface by combining its three original components (phylogenetic profiles, transcription units, and gene fusions) together with genomic expression and genome-wide dataset of protein-protein interaction discovered via text mining from PubMed abstract, and so forth. Putative protein-protein interaction of the PPI can be evaluated with the confidence score of functional association between two proteins across those genome-wide datasets. Different datasets are weighted differently for their respective contribution to the confidence score. In the STRING project, a weight was assigned to each dataset by benchmarking the performance of the prediction in this dataset against a common reference set of trusted knowledge. The developers chose the functional grouping of proteins maintained at KEGG (Kyoto Encyclopedia of Genes and Genomes) as the common reference set. The benchmark weight of each dataset in STRING corresponded to the probability of finding the linked proteins that were predicted in this dataset within the same KEGG pathway. In the equation of the confidence score, the confidence score is taken as *S*, the weight of each dataset is taken as *S*_*i*_, and *i* is the number of qualfied datasets with incidence of the pairwise connection. Therefore, the confidence score of the putative protein-protein interaction is evaluated through qualifying the naive Bayesian probability of the incidence of the corresponding protein connection across those multiple datasets under the assumption of independence of the various datasets. Larger confidence scores indicate higher confidence in a functional protein-protein association:

(2) $$\begin{array}{c} S=1-\prod_{i}^{}\left(1-S_{i}\right), \end{array}$$

where *S*_*i*_ is the weight assigned to each dataset over the common reference set.

**Figure 2:**
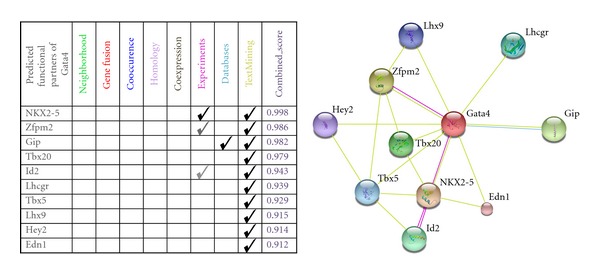
STRING search results for Gata4 from different sources. The network figure is the protein-protein interaction image from the search results (*Mus musculus*). Higher scores indicate greater confidence in the putative interaction. Here the highest confidence is given to NKX2-5 as an interactive partner of Gata4, as this is supported with experimental evidence.

Figure 2 shows an example result of a STRING query (http://STRING-db.org/) of the protein-protein interactions seeded by Gata4, a well-known transcription factor in cardiac development.

The confidence score for each putative protein-protein interaction is, therefore, a Bayesian-probability-like score supported by several types of genome-wide datasets. Putative PPI thus follows the evaluation in the genome-wide scope to gain confidence. 

**Figure 3:**
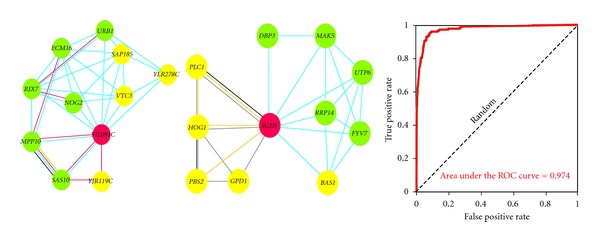
Predicted ribosome biogenesis genes are labeled as red nodes. Green nodes are the known ribosome biogenesis genes, and yellow nodes are genes that are not related to the ribosome biogenesis. Edge color indicates coexpression (light blue), affinity purification (red), yeast two-hybrid assay (green), genetic interaction (yellow), cocitation (gray), and literature curation (black). The ROC curve shows cross-validated recovery of the known ribosome biogenesis genes based on their network connectivity to one another. (This open-access figure was reproduced from Li et al., [25].)

Another approach applies the Bayesian schema to rationally extend the ribosome biogenesis pathway in yeast [25]. Li et al. constructed a computational predictor for inferring the ribosome biogenesis genes by integrating multiple heterogeneous datasets into a probabilistic model. This model employed a naive Bayesian probabilistic scoring system to integrate the multiple genome-wide datasets, including genomic expression, a genome-wide dataset of protein-protein interactions derived from literature curation, a genome-wide dataset of high-throughput yeast two-hybrid assays, a genome-wide dataset of affinity purification coupled with mass spectrometry, a genomic interaction dataset, and *in silico* genome-wide interaction datasets into a network (Figure 3). The plausibility that a putative yeast gene belongs to the ribosome biogenesis pathway was evaluated by calculating the naive Bayesian probability of the incidence of its association with the known ribosome biogenesis genes in the pathway. The ROC plot from cross-validation was employed to check the effectiveness of this schema (Figure 3). The top-scoring 212 genes were manually selected for the further experimental validation.

Bayesian schemas that apply the naive Bayesian probability are a powerful approach for analytic integration. Their application in the improves network construction in the examples given by evaluating the putative network with multiple genome-wide datasets integrated to calculate the confidence score. This schema always outperforms non-integration strategies. For example, the application of the Bayesian schema in an algorithm called MAGIC, as compared with the expression-based clustering methods, predicted more true positives than clustering methods did relative to the number of false positives [26]. 

#### 2.3.2. Supervised Learning

Supervised learning assumes that partial information is known for predictor variables and outcomes, and this partial information is leveraged to make deeper inferences of the target hypothesis. The known information is taken as the prior knowledge. Supervised approaches in statistics have been developed to make new inferences with the prior knowledge of the study objective to be integrated with the other relevant datasets. The accuracy of inferences regarding network topology is positively correlated with the amount of accurate prior knowledge. In contrast, unsupervised approaches have the problem that they are more likely to predict associations that are unreliable. The supervised learning schema can make inferences less error-prone. One analytic integration approach uses supervised learning to integrate the prior knowledge of the PPI with the other relevant genome-wide datasets to improve the effectiveness of PPI construction. 

Kato et al. developed a schema for supervised learning of yeast PPI using known protein-protein interactions as a prior knowledge to be integrated with the other relevant genome-wide datasets [27]. In this supervised network construction, a kernel matrix is applied as the basis of the integration. A kernel matrix is a matrix of similarity, and edges in the kernel-based network are assigned to the connected nodes whose kernel values (similarity) are above a certain threshold *δ*. The kernel matrix representation is an appropriate method for supervised PPI construction, as the network construction problem boils down to the problem of inferring an integrated kernel matrix of pairwise protein connections from combining the known yeast protein-protein interactions with the other relevant genome-wide datasets. Here, Kato et al. generated 3 main steps of the yeast PPI construction applying supervised learning.

**Step 1**

They translated the prior knowledge (known part of yeast PPI) into the kernel matrix by diffusion kernels. Diffusion kernels are functions for processing the network structure to mine the underlying relationships between nodes in the kernel matrix. However, this resulted in a regional kernel matrix of pairwise protein connections given a genome-wide scope because only the pairwise kernel values (the intensity of pairwise protein associations) of the proteins that were in the known part of PPI could be reconstructed. The regional kernel matrix could approximately recover the known PPI when the appropriate threshold *δ* of the kernel value was applied.

**Step 2**

A genome-wide dataset (e.g., genomic expression) with the same objective can be used to establish a new kernel matrix. Kato et al. took multiple types of genome-wide datasets into consideration for the PPI construction. They combined these new generated kernel matrices, each of which was calculated from a particular genome-wide dataset, such as genomic expression and genome-wide phylogenetic profiles, into a combined kernel matrix of pairwise protein associations in yeast.

**Step 3**

They integrated the combined kernel matrix with the regional kernel matrix of the known part of yeast PPI to infer the integrated kernel matrix of pairwise protein connections that offered the pairwise kernel values in the genome-wide scope to be able to qualify the PPI edges via comparing the kernel values against the threshold *δ*.

Accuracy of edge prediction was measured by a 10-fold cross-validation. With setting the parameter of the degree of kernel diffusion to 3.0 when translating the known PPI into the kernel matrix by diffusion kernels, the ROC score was 0.929 for the inferred yeast PPI. 

The supervised learning improves the PPI construction via integrating the experimentally-proven evidence of the study objective as the supervisor into the analysis of the other relevant genome-wide datasets. Thus the GRNs construction can also apply the schema of the supervised learning via having the known transcription factor-target gene regulations as the prior knowledge to be integrated with the other expression-relevant genome-wide datasets. One study compared supervised methods with unsupervised methods for GRN construction and found that the supervised methods are more reliable than the unsupervised ones [28].

#### 2.3.3. Network Topology Applications

In recent decades, a large amount of experimental evidence about biological networks has been collected, and this was coupled with progress in elucidating the network topological features. Approaches that have contributed to these strides in network biology include scale-free networks, small world networks, adaptive motifs, feed-back motifs, “AND” and “OR” logic motifs, and modular networks. Therefore, a systematic effort utilizing the network topological features will be required and will benefit the effectiveness of network construction. Modularity is one of the most accepted network topological features of GRNs. The modularity of GRNs can be represented by gene module members that are co-regulated via shared transcription factors combinatorially binding their promoters. Therefore, members in such gene modules manifest coexpression. Genomic expression and the genome-wide transcription factor-DNA binding sites are thus, integrated into GRN construction by identifying coexpressed genes with conserved TF binding sites in their promoters [29, 30]. Two examples applying this integration schema to the inference of GRNs are discussed below. 

**Figure 4:**
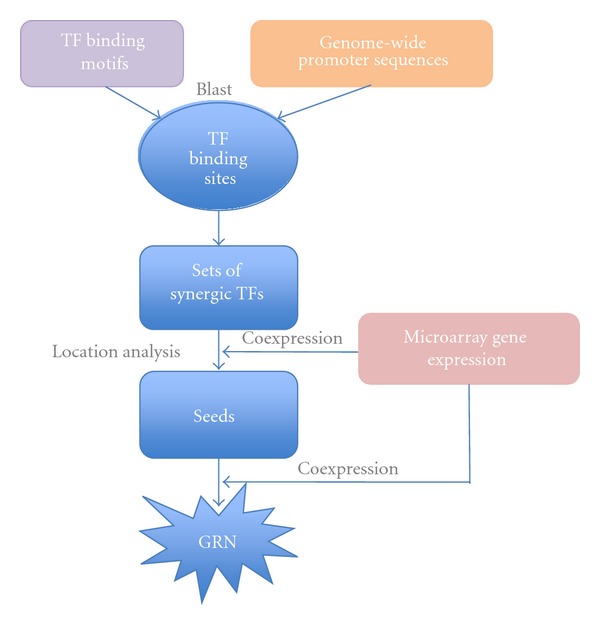
Workflow of the GRAM algorithm. Known TF binding motifs are blasted against the promoter sequences in the genome-wide scope to infer the corresponding TF binding sites. A set of synergic TFs is identified when the TFs' binding sites are close to each other. Regulated gene sets are defined by the corresponding sets of synergic TFs through location analysis. A “seed” of a gene module is selected from the regulated gene set based on the highly correlated expression. Then GRAM revisits the genomic expression to add more genes with closely correlated expression with the “seed” into the gene module of the “seed.” The GRN construction is fostered by the established regulations between the coexpression gene modules and their corresponding sets of synergic TFs.

GRAM is an algorithm for discovering GRNs by incorporating information from transcription factor (TF) binding motifs, genome sequence, and genomic expression [31]. Regulatory relationships are effectively identified by genome-wide location analysis of DNA-binding TFs via blasting the corresponding TF binding motifs against promoter sequences to infer the binding sites at the genome-wide scope. However, location analysis may infer potential physical interactions between TFs and DNA at the genome-wide scope but may not necessarily identify functional bindings. Integrating the location analysis with genomic expression, GRAM employs an effective and exhaustive strategy for GRN construction. It searches over all the possible combinations of TFs indicated by location analysis. When the binding sites are in close proximity, the corresponding TFs are defined to be in combination. A TF's combinations are used to identify its regulating gene set members that have common combinations of TFs binding their promoters as defined by location analysis. From the complete gene set, a subset is generated by members that have highly correlated expression in the expression dataset. The subset is taken as the “seed” of a gene module. Then GRAM revisits the genomic expression to add more genes having relatively high correlated expression with the “seed” into the gene module using less strict criteria (Figure 4). GRAM allows genes to belong to more than one module. Regulation is, therefore, inferred between the co-expression module and its TFs combination to foster GRN construction. 

In the GRAM project, this schema was applied to the TF binding motif data of 106 TFs and over 500 microarray expression experiments in *Saccharomyces cerevisiae*. The GRN was reconstructed via identification of modules. Gene modules were also identified as groups of genes annotated with similar pathways. Identified gene modules were controlled by more than one TF, which was the evidence for inferring the TFs' interactions (protein-protein interactions). GRAM can assign different regulators to genes with similar expression patterns, which cannot be accomplished using the expression clustering methods alone. Moreover, by applying the enrichment test of specific DNA binding motifs, genes in the discovered modules are more likely to be coregulated when compared with the set of genes obtained using genomic location analysis alone. 

**Figure 5:**
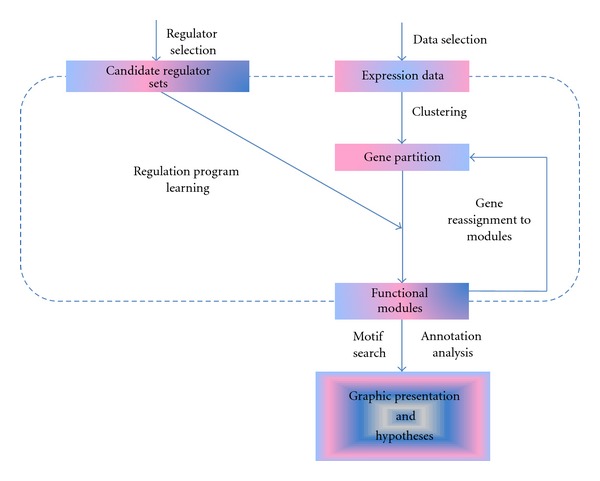
Workflow of the algorithm by Segal et al. This is an iterative procedure with application of the expectation maximization (EM) algorithm. In the maximization step (M step), genes are partitioned into modules that result from previous clustering upon genomic expression data and the best regulation program is learned for each module. In the E step, the best regulation programs corresponding are compared with each gene module to determine the optimal predictor (the optimal predictive regulation program). The module corresponding to the best predictor is selected and genes are reassigned to this module. The regulatory program learning stops on convergence. Secondly, TFs are associated with the regulatory module via an enrichment test of their corresponding binding motifs to the module.

Another application of this integration schema in GRNs construction was developed by Segal et al. [32]. Those authors designed an algorithm integrating a *Saccharomyces cerevisiae* genomic expression dataset with the genome-wide TF binding sites that were inferred via searching the corresponding TF binding motifs in the genome-wide scope. In their framework, a regulatory module was a set of genes that were regulated in concert by a shared regulation program. A regulation program specified the expression of the genes in the module as a function of the expression of a small set of regulators (Figure 5). After the enrichment test of TF binding motifs to the regulatory module, novel regulations were predicted between the TFs corresponding to the overrepresented binding motifs and the regulatory module to foster the GRN construction. Segal et al. found in many regulatory modules that the TFs corresponding to the overrepresented binding motifs of the module matched the known regulators of the genes in that module quite well.

Applying the modularity feature in GRNs construction via integrating genomic expression with genome-wide TF binding sites improves the quality of network construction. However, only limited information has been elucidated about the GRN topological features. The schema with GRN topology applied is expected to perform more compellingly with increasing knowledge of those features in GRN. 

#### 2.3.4. Choosing a Method of Analytic Integration

The Bayesian application schema for the naive Bayesian probability theorem is well accepted in most scientific fields. The naive Bayesian integrates multiple types of relevant genome-wide datasets into a scoring system that produces a confidence score for the inferred network (e.g., PPI and GRN). However, there is an important caveat with this approach: it is rational to apply the naive Bayesian theorem only when the situation satisfies the basic assumption that each type of source dataset is independent of any other. Therefore, under this assumption, there is no dependency between any two types. However, in reality some datasets have known dependencies. For example, in the case of STRING, the datasets of experiments, databases, and text mining are not completely independent of each other. The method of evaluating individual weight is also a controversial part of this schema. In the case of STRING, KEGG is used as the standard for calculating the weights. However, KEGG is an incomplete database in the genome-wide scope, and it is actually constructed from various experiments, databases, and text-mining resources, so it is necessarily dependent on those resources. It is, therefore, not a good standard, as it is biased—giving high weights to its own resources while giving low weights to the others. This may promote its accuracy but limit its predictive power. Hence, naïve Bayesian applications in GRN construction may be affected by those limitations. 

Supervised learning integrates prior knowledge of the study objective with the other relevant genome-wide datasets to learn the networks (e.g., PPI, GRN). However, the quality of its prediction varies with the quantity of the prior knowledge. Also, when multiple datasets are involved, weighting each dataset properly is still problematic. If we employ the nonweighting integration approach to make the primary prediction of the unknown part before it is trained by the prior knowledge, we may have better quality on the overall prediction even when the quantity of the prior knowledge is relatively small. 

The schema of network topology is a compelling strategy of GRN construction via integrating genomic expression with genome-wide TF binding sites. It associates the two sources through the modularity feature to connect the gene co-expression with the conserved TF binding sites on their promoters. However, as mentioned in the two examples, the TF binding sites are inferred from the corresponding TF binding motifs via a genome-wide blast. It will be improved when the CHIP-seq datasets regarding different TFs are employed instead to generate the genome-wide TF binding sites. It is a developing schema that keeps step with the development of our knowledge of network topological features. 

The schemas of supervised learning and network topology application may be described as advanced forms of the schema of Bayesian application, progressing from the naive to evidence-based logic. These approaches use principled and logical integration of datasets rather than integration only. Along with the increased experimentally proven knowledge about regulatory relationships, the schema of network topology application can be combined with supervised learning to gain increased confidence in the inferred GRNs. Overall, a positive-feedback effect that contributes to better GRNs helps to develop our knowledge of additional GRN topological features, while the more topological features provide more or better clues for GRNs' construction. The PPI could be embedded into the GRN to assess the TFs' combinatory regulations. 

## 3. Summary and Future Directions

GRN construction via integration of multiple types of genome-wide datasets or via literature-based information about regulation as the prior knowledge partly avoids or overcomes the drawbacks of the nonintegration strategies. Along with the continuous increase in the availability of new data sources, new opportunities emerge for us to use integration strategies to construct GRNs. There are two main categories of integration strategies: *structural integrations* for extracting and recombining the required datasets and *analytic integrations* for processing the queried datasets to infer GRNs. There are three main types of structural integration: *warehouse integration* naively aggregates the required datasets into local storage before data querying, *mediator-based integration* establishes a mediator application to retrieve the required datasets via reformatting the user's query into the formats of queries in local data sources at the time of data processing, and the *navigational integration* follows the chain of data querying at the time of data processing via using the query outputs of one step in the process as query inputs in a next step. In a subsequent analytic integration, the schema of Bayesian applications use the naive Bayesian probability to integrate multiple types of genome-wide datasets into a scoring system to compute a confidence score for inferred GRNs. Supervised learning integrates the prior knowledge of the study objective with the other relevant genome-wide datasets to learn the GRNs. And the schema of network topology applications integrate genomic expression with genome-wide TF binding sites through the modularity feature to connect gene co-expression with conserved TF binding sites in their promoters to foster the GRNs construction. Overall, the integration strategies perform well and reliably as compared to the non-integration strategies. Structural integration and analytic integration take central roles in the overall integration strategy of GRN construction. 

Recently, cooperation of traditional experimental approaches with computational approaches has energized biomedical research. These new approaches offer the ability to computationally infer novel hypotheses from prior knowledge and relevant datasets to guide experimentation by setting research priorities. A salient example of this successful cooperation defines how to rationally extend the ribosome biogenesis pathway in yeast [25]. After revealing 212 candidates from the Bayesian applied integration analysis of multiple relevant genome-wide datasets, experiments were employed to validate their findings. Li et al. identified 15 previously unreported ribosome biogenesis genes (TIF4631, SUN66, YDL063C, JIL5, TOP1, SGD1, BCP1, YOR287C, BUD22, YIL091C, YOR006C/TSR3, YOL022C/TSR4, SAC3, NEW1, and FUN1). Segal et al. used a similar workflow to validate the GRN construction [32]. Therefore, GRNs inferred from the analysis with multiple types of integrated datasets offer a sophisticated atlas for setting research priorities. 

## Figures and Tables

**Table 1:** Prominent databases.

Category	Databases
Metabolic pathways	KEGG, ENZYME
Signaling pathways	KEGG, WikiPathways
Protein-protein interactions	BIND, STRING
Transcription factor binding motifs	JASPAR, TRANSFAC
Genetic interaction networks	BIND, BioGRID
Gene expression	GEO, ArrayExpress
Sequences	UCSC Genome Browrer
Protein-compound interactions	DrugBank, STITCH, ResNet, CLiBE
Gene-disease associations	OMIM

**Table 2:** Properties of distinctive structural integration approaches.

Approach	Maintenance	System stability	Effectiveness
Warehouse	Difficult, costly	Stable	Poor
Mediator-based	Easy for LAV	Depends on source availability, accessibility, traffic	Fair
Navigational	Easy	Depends on source availability, accessibility, traffic	Good

## Abbreviations

BIC: Bayesian Information Criteria
EMBL: European Molecular Biology Laboratory
GO: Gene ontology
GRN: Gene regulatory network
KEGG: Kyoto Encyclopedia of Genes and Genomes
NAR: Nucleic Acids Research
NCBI: National Center for Biotechnology Information
PCR: Polymerase chain reaction
PPI: Protein-protein interaction
SNP: Single-nucleotide polymorphism.

### Glossary

Bayesian information criterion (BIC): In statistics, it is a criterion for model selection among a finite set of models
Cis-regulatory motif: A nucleotide pattern that is widespread and has a biological significance for regulatory factor binding
ChIP-seq: a technology that combines chromotin immunoprecipitation (ChIP) with massive sequencing to identify the binding sites of DNA-associated proteins on a genomic scale
Cross-validation: A technique for assessing how the results of a statistical analysis will generalize to an independent data set
Epistatic: In genetics, it is the phenomenon where the effects of one gene are modified by one or several other genes
Gene ontology: A controlled vocabulary for annotating genes and gene products
Gene regulatory network: A network that summarizes gene regulatory influences in a biological process
*In silico*: ALatin expression used to mean “performed on computer” or “computer simulation”
Mesoderm: In all bilaterian animals, the mesoderm is one of the three primary germ cell layers in the early embryo
Operon: In genetics, an operon is a functioning unit of genomic DNA containing a cluster of genes under the control of a single regulatory signal or promoter
Phylogenetic profile: Also called phylogenetic tree, is a branching diagram or “tree” showing the inferred evolutionary relationships among various biological species or other entities based upon similarities and differences in their physical and/or genetic characteristics
Schema: A representation of a plan, theory, or data structure, normally expressed as an outline or model
Semantic: Relating to meaning language or logic; in a biological context, this usually refers to the meaning of specifically defined annotations, concepts, or logical relationships between biological entities
Wrapper: A computer program that translates one format of data to another or a computer program that simplifies user interactions with a more complex program.

## References

[1] Davidson EH, Levine MS (2008). Properties of developmental gene regulatory networks. *Proceedings of the National Academy of Sciences of the United States of America*.

[2] Levine M, Davidson EH (2005). Gene regulatory networks for development. *Proceedings of the National Academy of Sciences of the United States of America*.

[3] Li E, Davidson EH (2009). Building developmental gene regulatory networks. *Birth Defects Research Part C*.

[4] Erwin DH, Davidson EH (2002). The last common bilaterian ancestor. *Development*.

[5] Davidson EH, Rast JP, Oliveri P (2002). A genomic regulatory network for development. *Science*.

[6] Calva D, Dahdaleh FS, Woodfield G (2011). Discovery of SMAD4 promoters, transcription factor binding sites and deletions in juvenile polyposis patients. *Nucleic Acids Research*.

[7] Zaret KS, Liu JK, DiPersio CM (1990). Site-directed mutagenesis reveals a liver transcription factor essential for the albumin transcriptional enhancer. *Proceedings of the National Academy of Sciences of the United States of America*.

[8] Smith J, Theodoris C, Davidson EH (2007). A gene regulatory network subcircuit drives a dynamic pattern of gene expression. *Science*.

[9] de Jong H (2002). Modeling and simulation of genetic regulatory systems: a literature review. *Journal of Computational Biology*.

[10] Faith JJ, Hayete B, Thaden JT (2007). Large-scale mapping and validation of Escherichia coli transcriptional regulation from a compendium of expression profiles. *PLoS Biology*.

[11] Margolin AA, Nemenman I, Basso K (2006). ARACNE: an algorithm for the reconstruction of gene regulatory networks in a mammalian cellular context. *BMC Bioinformatics*.

[12] Madar A, Greenfield A, Vanden-Eijnden E, Bonneau R (2010). DREAM3: network inference using dynamic context likelihood of relatedness and the inferelator. *PloS One*.

[13] Zoppoli P, Morganella S, Ceccarelli M (2010). TimeDelay-ARACNE: reverse engineering of gene networks from time-course data by an information theoretic approach. *BMC Bioinformatics*.

[14] Gerstein MB, Kundaje A, Hariharan M (2012). Architecture of the human regulatory network derived from ENCODE data. *Nature*.

[15] Hernandez T, Kambhampati S (2004). Integration of biological sources: current systems and challenges ahead. *Sigmod Record*.

[16] Stein LD (2003). Integrating biological databases. *Nature Reviews Genetics*.

[17] Fujita PA, Rhead B, Zweig AS (2011). The UCSC Genome Browser database: update 2011. *Nucleic Acids Research*.

[18] Arrais J, Pereira JE, Fernandes J, Oliveira JL (2009). GeNS: a biological data integration platform. *Proceedings of World Academy of Science, Engineering and Technology*.

[19] Dias GS, Oliveira JL, Vicente J, Martin-Sanchez F (2006). Integrating medical and genomic data: a successful example for rare diseases. *Studies in Health Technology and Informatics*.

[20] Jensen LJ, Kuhn M, Stark M (2009). STRING 8—a global view on proteins and their functional interactions in 630 organisms. *Nucleic Acids Research*.

[21] Snel B, Lehmann G, Bork P, Huynen MA (2000). STRING: a web-server to retrieve and display the repeatedly occurring neighbourhood of a gene. *Nucleic Acids Research*.

[22] von Mering C, Huynen M, Jaeggi D, Schmidt S, Bork P, Snel B (2003). STRING: a database of predicted functional associations between proteins. *Nucleic Acids Research*.

[23] von Mering C, Jensen LJ, Kuhn M (2007). STRING 7—recent developments in the integration and prediction of protein interactions. *Nucleic Acids Research*.

[24] von Mering C, Jensen LJ, Snel B (2005). STRING: known and predicted protein-protein associations, integrated and transferred across organisms. *Nucleic Acids Research*.

[25] Li Z, Lee I, Moradi E, Hung NJ, Johnson AW, Marcotte EM (2009). Rational extension of the ribosome biogenesis pathway using network-guided genetics. *PLoS Biology*.

[26] Troyanskaya OG, Dolinski K, Owen AB, Altman RB, Botstein D (2003). A Bayesian framework for combining heterogeneous data sources for gene function prediction (in Saccharomyces cerevisiae). *Proceedings of the National Academy of Sciences of the United States of America*.

[27] Kato T, Tsuda K, Asai K (2005). Selective integration of multiple biological data for supervised network inference. *Bioinformatics*.

[28] Cerulo L, Elkan C, Ceccarelli M (2010). Learning gene regulatory networks from only positive and unlabeled data. *BMC Bioinformatics*.

[29] Pavesi G, Mereghetti P, Mauri G, Pesole G (2004). Weeder Web: discovery of transcription factor binding sites in a set of sequences from co-regulated genes. *Nucleic Acids Research*.

[30] Pavesi G, Pesole G (2006). Using Weeder for the discovery of conserved transcription factor binding sites. *Current Protocols in Bioinformatics*.

[31] Bar-Joseph Z, Gerber GK, Lee TI (2003). Computational discovery of gene modules and regulatory networks. *Nature Biotechnology*.

[32] Segal E, Shapira M, Regev A (2003). Module networks: identifying regulatory modules and their condition-specific regulators from gene expression data. *Nature Genetics*.

